# The transcription factor Gli3 promotes B cell development in fetal liver through repression of Shh

**DOI:** 10.1084/jem.20160852

**Published:** 2017-07-03

**Authors:** Anisha Solanki, Ching-In Lau, José Ignacio Saldaña, Susan Ross, Tessa Crompton

**Affiliations:** 1 Great Ormond Street Institute of Child Health, University College London, London, England, UK; 2 School of Health, Sport, and Bioscience, University of East London, London, England, UK

## Abstract

Before birth, B cells develop in the fetal liver (FL). In this study, we show that Gli3 activity in the FL stroma is required for B cell development. In the Gli3-deficient FL, B cell development was reduced at multiple stages, whereas the Sonic hedgehog (Hh [Shh])–deficient FL showed increased B cell development, and Gli3 functioned to repress *Shh* transcription. Use of a transgenic Hh-reporter mouse showed that Shh signals directly to developing B cells and that Hh pathway activation was increased in developing B cells from Gli3-deficient FLs. RNA sequencing confirmed that Hh-mediated transcription is increased in B-lineage cells from Gli3-deficient FL and showed that these cells expressed reduced levels of B-lineage transcription factors and B cell receptor (BCR)/pre-BCR–signaling genes. Expression of the master regulators of B cell development Ebf1 and Pax5 was reduced in developing B cells from Gli3-deficient FL but increased in Shh-deficient FL, and in vitro Shh treatment or neutralization reduced or increased their expression, respectively.

## Abstract (precis)

Solanki et al. show that stromal activity of the transcription factor Gli3 is required for B cell development in the fetal liver. Gli3 functions to repress Shh expression, and Shh signals to developing B cells to regulate their development at multiple developmental stages.

## Introduction

During B cell development in the fetal liver (FL), hematopoietic stem cells, defined as lineage-negative Sca-1^+^ c-Kit^+^ cells, mature to gain expression of IL-7Rα (CD127) and are known as common lymphoid progenitor (CLP) cells (Egawa et al., 2001; Mebius et al., 2001). However, this population is multipotent and still contains cells with potential for both lymphoid and myeloid lineages (Mebius et al., 2001). Commitment to the B cell lineage first occurs on embryonic day 12.5 (E12.5), as cells mature to initially express CD19 or B220 and are defined as either B-1 (CD19^+^B220^lo-neg^) or B-2 (CD19^−^B220^+^) lineage cells (Egawa et al., 2001; Dorshkind and Montecino-Rodriguez, 2007; Montecino-Rodriguez and Dorshkind, 2012). B-1 cell development is more prominent in the FL and fetal BM, whereas B-2 cells are mainly produced in the adult BM (Montecino-Rodriguez and Dorshkind, 2012).

Both B-1 and B-2 progenitors mature into B220^+^CD19^+^ double-positive cells, which undergo immunoglobulin heavy chain gene rearrangement to give rise to the first cells that express cell surface µH (pre-BCR; Dorshkind and Montecino-Rodriguez, 2007; Montecino-Rodriguez and Dorshkind, 2012). This pre–B cell population can also be identified by cell surface expression of BP-1, before rearrangement of the light chain locus and cell surface expression of IgM (Hardy and Hayakawa, 2001; Dorshkind and Montecino-Rodriguez, 2007; Montecino-Rodriguez and Dorshkind, 2012).

Here, we investigate the role of Sonic hedgehog (Hh [Shh]) and the transcription factor Gli3 in the regulation of B cell development in the FL. Shh is one of three mammalian Hh proteins (Shh, Indian Hh [Ihh], and Desert Hh [Dhh]) that share a common signaling pathway (Ingham et al., 2011; Ramsbottom and Pownall, 2016). Hh proteins signal by binding to their cell surface receptor Patched1 (Ptch1), thereby releasing Ptch1’s repression of Smoothened (Smo), allowing Smo to transduce the Hh signal. At the end of the signaling pathway are the Hh-responsive transcription factors Gli1, Gli2, and Gli3 (Ingham et al., 2011). *Gli1* is itself an Hh-target gene and encodes an activator of transcription (Park et al., 2000), whereas Gli2 and Gli3 can be processed to function as transcriptional activators (Gli2A/Gli3A, in the presence of Hh pathway activation) or transcriptional repressors (Gli2R/Gli3R, in the absence of Hh pathway activation; Sasaki et al., 1999). Gli2 is required to initiate the Hh signal and functions largely as a transcriptional activator in vivo (Park et al., 2000; Bai et al., 2002). In contrast, Gli3 functions predominantly as a transcriptional repressor in vivo (Wang et al., 2000). The pathway has multiple positive and negative feedback mechanisms, and *Ptch1* is itself an Hh-target gene, functioning to sequester Hh proteins and limit activation of the pathway (Ingham et al., 2011).

Gli3 can have both Hh-independent and Hh-dependent functions (te Welscher et al., 2002; Hager-Theodorides et al., 2009). Gli3R functions to limit Hh pathway activation in many tissues (Wang et al., 2000; Ahn and Joyner, 2004; Hager-Theodorides et al., 2009). There are at least two distinct mechanisms by which Gli3R can limit Hh signaling: it may repress expression of Hh genes in the Hh-producing cell via repression of Hh-activating genes, thus limiting Hh protein concentration in the tissue. For example, during prepatterning of the limb bud, Gli3R spatially limits the expression of dHand, an activator of *Shh* gene expression (te Welscher et al., 2002). Alternatively, when Gli3 is expressed in the signal-receiving cell, the concentration of Gli3R in a given cell increases the further away the cell is located from the Hh-secreting source, resulting in correspondingly increased repression of Hh-target genes (Wang et al., 2000; te Welscher et al., 2002). In fact, in many tissues, such as thymus and limb bud, Shh and Gli3 have opposing functions, with Shh deficiency and Gli3 deficiency giving opposite phenotypes (Wang et al., 2000; Shah et al., 2004; Hager-Theodorides et al., 2005, 2009; Barbarulo et al., 2016). Here, we show that Gli3 expressed in the FL stroma promotes B-lineage commitment of hematopoietic progenitor cells and B cell development by suppression of Shh signaling.

## Results

### Impaired B-lineage commitment in the Gli3-deficient FL

**Figure 1.**
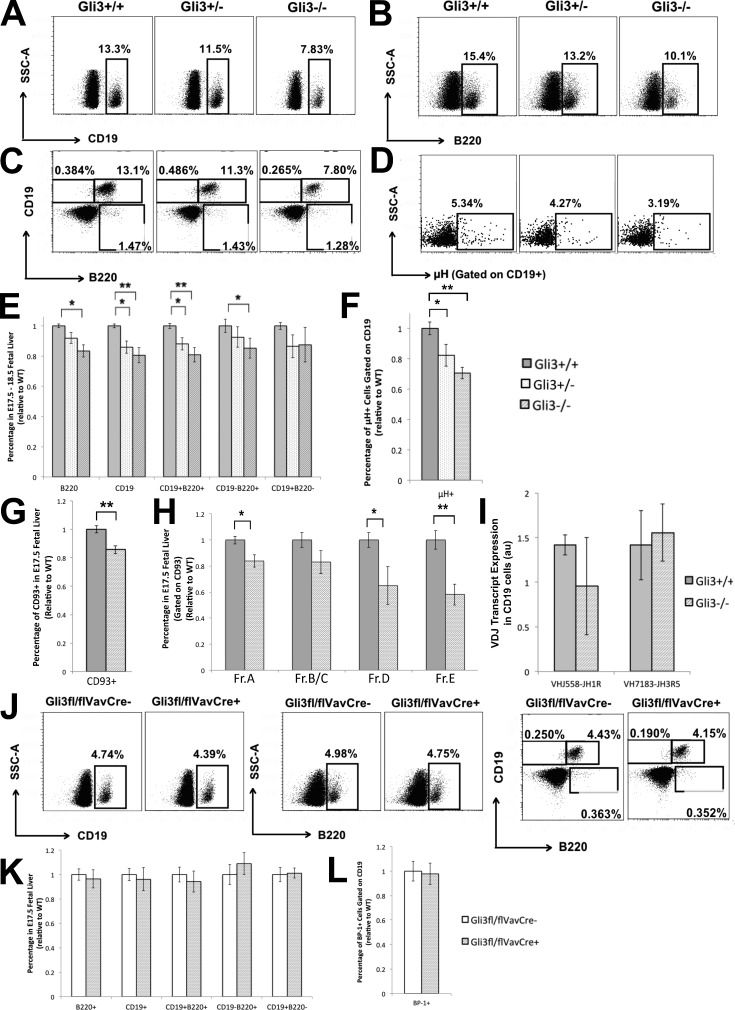
**B lineage development in E18.5 and E17.5 Gli3^+/+^, Gli3^+/−^, and Gli3^−/−^ and E17.5 *Gli3^fl/fl^*VavCre^+^ FL.** (A–F) Flow cytometry profile of E18.5 FL from Gli3^+/+^ (WT), Gli3^+/−^, and Gli3^−/−^ littermates after red blood cell lysis from a representative experiment. (A–D) Dot plots: SSC against CD19 (A), SSC against B220 (B), CD19 versus B220 (C), and SSC against μH gated on CD19^+^ cells (D). (E and F) Bar charts: mean percentage of FL populations, relative to mean of WT littermates ± SEM, giving statistical significance by Student’s *t* test compared with WT littermate FL for: B220^+^ (Gli3^−/−^, P = 0.02), CD19^+^ (Gli3^+/−^, P = 0.03; Gli3^−/−^, P = 0.002), CD19^+^B220^+^ (Gli3^+/−^, P = 0.05; Gli3^−/−^, P = 0.001), CD19^−^B220^+^ (Gli3^−/−^, P = 0.02), CD19^+^B220^−^ (E) and µH gated on CD19^+^ cells (Gli3^+/−^, P = 0.05; Gli3^−/−^, P = 0.001), from E17.5 and E18.5 Gli3^+/−^ (*n* = 25), Gli3^−/−^ (*n* = 14), and Gli3^+/+^ (*n* = 15). (G and H) Bar chart: mean ± SEM of FL populations, relative to mean of WT littermates, giving statistical significance by Student’s *t* test for Gli3^−/−^ (*n* = 4) and WT (*n* = 3), where G shows percentage of CD93^+^ cells (Gli3^−/−^, P = 0.008) and H shows four populations, gated on CD93^+^. For fraction A (Fr.A): Gr1^−^, Mac1^−^, Ter119^−^, CD71^−^, ckit^+^, and CD127^+^ (P = 0.03); fraction B/C: CD19^+^, HSA^+^, CD43^+^, and BP-1^−^; fraction D: CD19^+^, HSA^+^, CD43^+^, and BP-1^+^ (P = 0.03); and fraction E: CD19^+^HSA^+^μH^+^ (P = 0.002). (I) Bar chart: mean ± SEM of Q-RT-PCR analysis of VDJ recombination in FACS-sorted FL CD19^+^ cells from WT (*n* = 4) and Gli3^−/−^ (*n* = 4) littermates, normalized relative to HS5 primers for two different VDJ recombination transcripts: VHJ558-Fw and JH1R, and VH7183-Fw and JH3R5. au, arbitrary units. (J) Flow cytometry profile of E17.5 FLs from control (*Gli3^fl/fl^*VavCre^−^) and Gli3coKo (*Gli3^fl/fl^*VavCre^+^) littermate embryos from a representative experiment. (K and L) Bar charts: mean percentage, relative to mean of WT littermates ± SEM, of B220^+^, CD19^+^, CD19^+^B220^+^, CD19^−^B220^+^, CD19^+^B220^−^ (K) and BP-1^+^ gated on CD19^+^ cells (L), from Gli3coKO (*Gli3^fl/fl^*VavCre^+^; *n* = 8) and control (*Gli3^fl/fl^*VavCre^−^; *n* = 8) littermates. There were no significant differences by Student’s *t* test. *, P ≤ 0.05; **, P ≤ 0.01.

The Hh-responsive transcription factor Gli3 is expressed in mouse FL (Cridland et al., 2009) and human FL and BM (Fig. S1 A; Su et al., 2004). Microarray expression profiles from the Immgen database show expression of *Smo*, *Ptch1*, and the *Gli* transcription factors in mouse E15 FL hematopoietic stem cells, CLPs, and pro–B (CD19^+^µH^−^) and pre–B (CD19^+^µH^+^) cells (Fig. S1, B–F; Heng et al., 2008). Therefore, to investigate the role of Gli3 in fetal B cell development, we analyzed B-lineage markers in Gli3^−/−^, Gli3^+/−^, and Gli3^+/+^ (WT) littermate E18.5 FL. We found statistically significant gene dose–dependent decreases in the proportions of the CD19^+^ cells, B220^+^ cells, and CD19^+^B220^+^ cells in the E18.5 *Gli3*-mutant FL (Fig. 1, A–E). The Gli3^−/−^ FL also showed a significant decrease in the proportion of CD19^−^B220^+^ B2 progenitor cells (Fig. 1, C and E). Additionally, the proportion of CD19^+^ cells that expressed the cell surface heavy chain µH was significantly reduced in the *Gli3*-mutant FL compared with WT (Fig. 1, D and F).

We further characterized early B cell development by staining against the B-lineage marker CD93 and then subdividing the CD93^+^ population by expression of ckit, CD127, heat stable antigen (HSA), CD43, and BP-1, in addition to CD19 and µH expression, to identify four fractions of increasing maturity (Fig. 1, G and H). The overall proportion of CD93^+^ cells (B lineage committed) was significantly reduced in the Gli3^−/−^ FL compared with WT (Fig. 1 G). Gating on these CD93^+^ cells, the proportion of the early ckit^+^CD127^+^ population (fraction A) was also significantly reduced in the Gli3^−/−^ FL compared with WT, as were the later CD43^+^CD19^+^HSA^+^BP-1^+^ (fraction D) and CD19^+^HSA^+^µH^+^ populations (fraction E; Fig. 1 H).

As Gli3 deficiency influenced early B cell maturation and reduced the proportion of µH^+^ B-lineage cells, we used quantitative RT-PCR (Q-RT-PCR) to test whether heavy chain rearrangements were reduced in FACS-sorted CD19^+^ cells from Gli3^−/−^ and WT littermates. We quantified rearrangements between two different VH to JH segments and found no evidence for reduced gene rearrangement in developing B cells from the Gli3-deficient FL (Fig. 1 I).

### Gli3 activity is not required for B cell development in the hematopoietic compartment of the FL

To investigate whether the reduction in B cell development in the *Gli3*-mutant FL is caused by cell-intrinsic Gli3 activity in the hematopoietic compartment or caused by Gli3 activity in the nonhematopoietic compartment (stroma), we used the Cre-loxP system to conditionally delete *Gli3* from the hematopoietic lineage in *Gli3^fl/fl^* VavCre^+^ (Gli3coKO) embryos. We found no significant difference in B cell differentiation between the control (Cre negative) and Gli3coKO FL, and the proportion of cells that expressed CD19, B220, and BP-1 was not different between control and Gli3coKO (Fig. 1, J–L). Therefore, Gli3 activity in the FL stroma, rather than hematopoietic cell–intrinsic activity, regulates B cell differentiation.

### Increased Hh signaling in the *Gli3*-mutant FL

**Figure 2.**
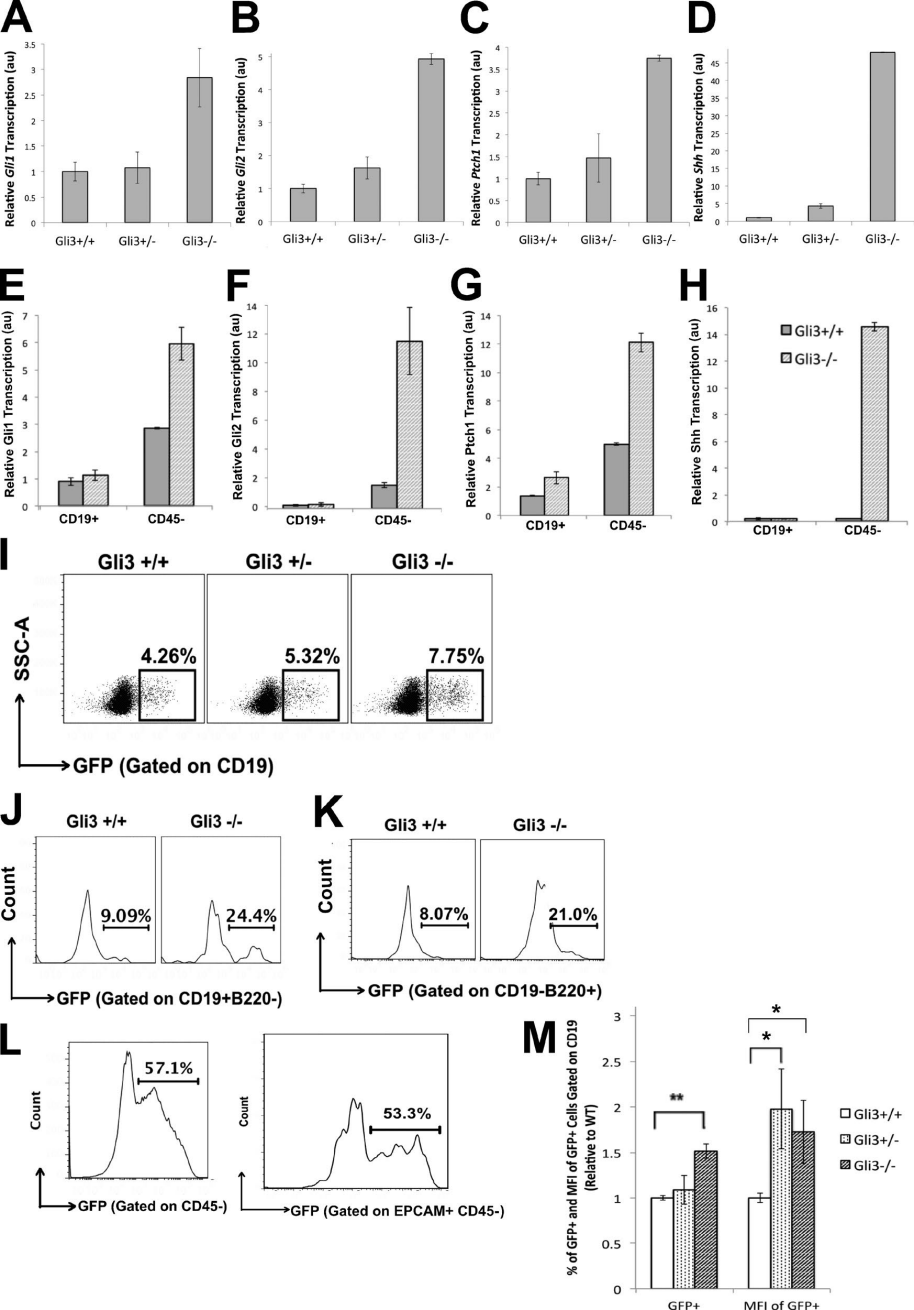
**Expression of Hh pathway components and active Hh signaling in E14.5 and E17.5 WT and *Gli3*-mutant FL.** (A–H) Bar charts: Representative experiments show mean ± SEM (*n* = 3) of Q-RT-PCR analysis from WT, Gli3^+/−^, and Gli3^−/−^ littermates for whole FL *Gli1* (A), *Gli2* (B), and *Ptch1* (C) on E17.5 and *Shh* on E14.5 (D) and for FACS-sorted CD19^+^ and CD45^−^ (stromal) cells for *Gli1* (E), *Gli2* (F), *Ptch1* (G), and *Shh* (H). au, arbitrary units. (I–M) Flow cytometric analysis of GFP expression in GBS-GFP–reporter transgenic E17.5 FL from WT, Gli3^+/−^, and Gli3^−/−^ littermates. (I) Dot plots: SSC versus GFP fluorescence, gated on CD19^+^ cells. (J and K) Histograms: expression of GFP in CD19^+^B220^−^ (J) and CD19^−^B220^+^ (K) in the Gli3^+/+^ and Gli3^−/−^ E17.5 FL. (L) Histograms: expression of GFP in the E17.5 WT FL stroma (CD45^−^; left) and CD45–epithelial cell adhesion molecule (EPCAM)^+^ stromal cells (right). (M) Bar charts: mean percentage and mean MFI ± SEM of GFP fluorescence in CD19^+^ cells, relative to the mean of WT. Gli3^+/−^, *n* = 4; Gli3^−/−^, *n* = 3; WT, *n* = 3. Differences are statistically significant by Student’s *t* test, compared with WT; for percentage of GFP^+^ in the CD19^+^ cells, for Gli3^−/−^, P = 0.002, and for the MFI of GFP on the CD19^+^ cells, for Gli3^+/−^, P = 0.03 and, for Gli3^−/−^, P = 0.04. *, P ≤ 0.05; **, P ≤ 0.01.

As Gli3 can have Hh-independent and Hh-dependent functions and can act to limit Hh pathway activation, we tested whether the *Gli3*-mutant FL had increased levels of Hh pathway activation by measuring the transcription of Hh pathway components and target genes by Q-RT-PCR from the tissue (Fig. 2, A–D). The Hh-target genes, the transcription factor *Gli1*, and the Hh receptor *Ptch1* were increased in the Gli3^−/−^ FL compared with WT, as was the Hh-responsive transcription factor *Gli2* (Fig. 2, A–C). Transcription of *Shh* was also increased, consistent with Gli3 functioning to repress *Shh* expression (Fig. 2 D). Then, we FACS-sorted CD45^+^CD19^+^ (B lineage) and nonhematopoietic CD45^−^ (stroma) cells from Gli3^−/−^ and littermate WT FL and compared expression of Hh pathway components and target genes (Fig. 2, E–H). Expression of *Gli1* was higher in nonhematopoietic WT cells than in the CD19^+^ population, and its expression was increased in the Gli3-deficient populations, with greater increase in the nonhematopoietic (stromal) Gli3-deficient compartment (Fig. 2 E). *Gli2* expression was approximately fivefold higher in the stromal cells of the Gli3-deficient FL compared with WT and was relatively very low in the CD19^+^ fraction (Fig. 2 F). In contrast, expression of *Ptch1* was increased in both populations sorted from Gli3^−/−^ compared with WT FL (Fig. 2 G). Expression of *Shh* was greatly up-regulated in the nonhematopoietic (stromal) component of the Gli3^−/−^ FL compared with WT, consistent with Gli3 functioning to repress *Shh* expression in the FL stroma (Fig. 2 H) and with a previous study on expression of Shh by Dlk^+^ hepatoblasts (Hirose et al., 2009). Although Gli3 and Gli2 can have overlapping or redundant functions in some tissues, we found no evidence for redundancy between Gli3 and Gli2 in repression of Shh expression in the stroma; in the absence of *Gli3*, *Shh* was up-regulated.

Given that transcription of Hh-target genes and *Shh* were up-regulated in the absence of Gli3, whereas the B cell progenitor populations were reduced, we tested whether developing B cells and stromal cells are undergoing active Hh signaling in the FL. We used Gli binding site (GBS)–GFP-transgenic reporter mice, in which GFP is expressed when activator forms of Gli proteins bind to the GBS in the transgene, to measure active Hh-dependent transcription (Balaskas et al., 2012). Approximately 4% of CD19^+^ cells expressed GFP in the WT FL (Fig. 2 I), and a higher level of GFP expression of ∼8% and ∼9% was observed in the CD19^+^B220^−^ (B-1 progenitors) and CD19^−^B220^+^ (B-2 progenitors), respectively (Fig. 2, J–K), suggesting that Hh signaling is higher in cells transitioning from the immature B1 and B2 progenitor stages toward the CD19^+^B220^+^ stage. High proportions of nonhematopoietic (CD45^−^) stromal FL cells (∼57%) and of the epithelial cell adhesion molecule^+^ subset of CD45^−^ FL cells (∼53%) expressed GFP (Fig. 2 L), confirming the Q-RT-PCR data, which indicated that Hh signaling is also active in the nonhematopoietic stromal compartment (Fig. 2, E and G). The proportion of GFP-expressing CD19^+^, CD19^+^B220^−^, and CD19^−^B220^+^ cells was significantly increased in the Gli3^−/−^ compared with WT (Fig. 2, I–K and M). However, there was no significant difference in the proportion of GFP^+^ cells between Gli3^+/−^ and WT (Fig. 2 M). The mean fluorescence intensity (MFI) of the CD19^+^GFP^+^ cells significantly increased from WT to Gli3^+/−^ and from WT to Gli3^−/−^, indicating higher Hh-dependent transcription in individual cells (Fig. 2 M).

### Hh signaling is a negative regulator of fetal B cell development

**Figure 3.**
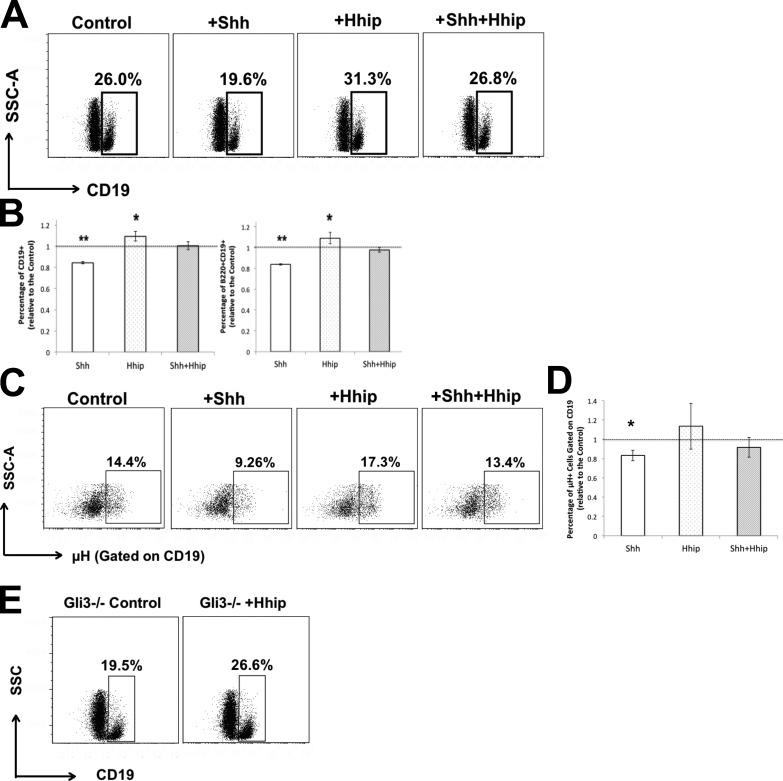
**Modulation of B cell development by rShh and rHhip treatment of WT and Gli3^−/−^ FLOCs.** (A–E) WT E17.5 FLOCs were treated with recombinant proteins, as stated, for 4 d and analyzed by flow cytometry. *n* = 4. (A) Dot plots: SSC versus CD19 staining in control (untreated) and treated with rShh, rHhip, and rShh + rHhip. (B) Bar charts: mean ± SEM, relative to the mean of control untreated FLOCs from littermates, showing statistical significance by Student’s *t* test compared with untreated for CD19^+^ cells (rShh treatment, P = 0.002; rHhip treatment, P = 0.04; left), and CD19^+^B220^+^ cells (rShh treatment, P = 0.002; rHhip treatment, P = 0.04; right). The dotted line indicates mean of untreated control WT. (C) Dot plots: µH^+^ gated on the CD19^+^ population in control (untreated) and treated with rShh, rHhip, and with rShh and rHhip together. (D) Bar chart: mean ± SEM, relative to the mean of control untreated FLOCs showing statistical significance by Student’s *t* test compared with untreated for µH^+^ gated on the CD19^+^ population (rShh treatment, P = 0.03). The dotted line indicates mean of untreated control WT. (E) Gli3^−/−^ FLOCs treated with rHhip and control untreated Gli3^−/−^ FLOCs were cultured for 4 d. Dot plots: SSC versus CD19 staining. Differences in the mean percentages were statistically significant between rHhip treatment and control untreated. P = 0.02 for CD19^+^ cells, and P = 0.03 for CD19^+^B220^+^ cells. *, P ≤ 0.05; **, P ≤ 0.01.

The Gli3^−/−^ FL had increased *Shh* transcription, increased expression of Hh-target genes, and increased GFP expression in developing B cells in the GBS-GFP reporter–transgenic embryos (Fig. 2). Thus, we tested whether increased Hh signaling reduces B cell development in vitro. We assessed B cell populations after 4 d in WT E17.5 FL organ cultures (FLOCs) treated with recombinant Shh (rShh) alone, with recombinant Hh-interacting protein (Hhip [rHhip]; to bind and neutralize endogenous Hh proteins in the cultures) alone, or treated with both rShh and rHhip, compared with control nontreated FLOCs (Fig. 3, A and B). Treatment with rShh significantly reduced the proportion of CD19^+^ cells from 26% in control cultures to 19.6% in rShh-treated FLOCs, whereas neutralization of endogenous Hh proteins by treatment with rHhip significantly increased the proportion of CD19^+^ cells to 31.3%. To confirm the specificity of the reagents and that the inhibitory effect of rShh was not the result of nonspecific toxicity, we added both reagents together and found that the proportion of CD19^+^ cells was not significantly different from control cultures (Fig. 3, A and B). The proportion of the B220^+^CD19^+^ and CD19^+^µH^+^ populations were also both significantly decreased compared with the control by rShh treatment and increased by rHhip treatment in FLOCs (Fig. 3, B–D). Therefore, to test whether the reduction in B cell development in the Gli3^−/−^ FL is caused by an increase in Hh proteins, we treated the Gli3^−/−^ FLOCs with rHhip to neutralize endogenous Hh proteins. The proportion of CD19^+^ cells was significantly increased compared with the untreated Gli3^−/−^ control (Fig. 3 E). This suggests that the reduction in B cell development in the Gli3 mutant was largely caused by an increase in Hh proteins in the FL and that Hh signaling negatively regulates B cell development in vitro.

### B cell development in the Shh^−/−^ fetus

**Figure 4.**
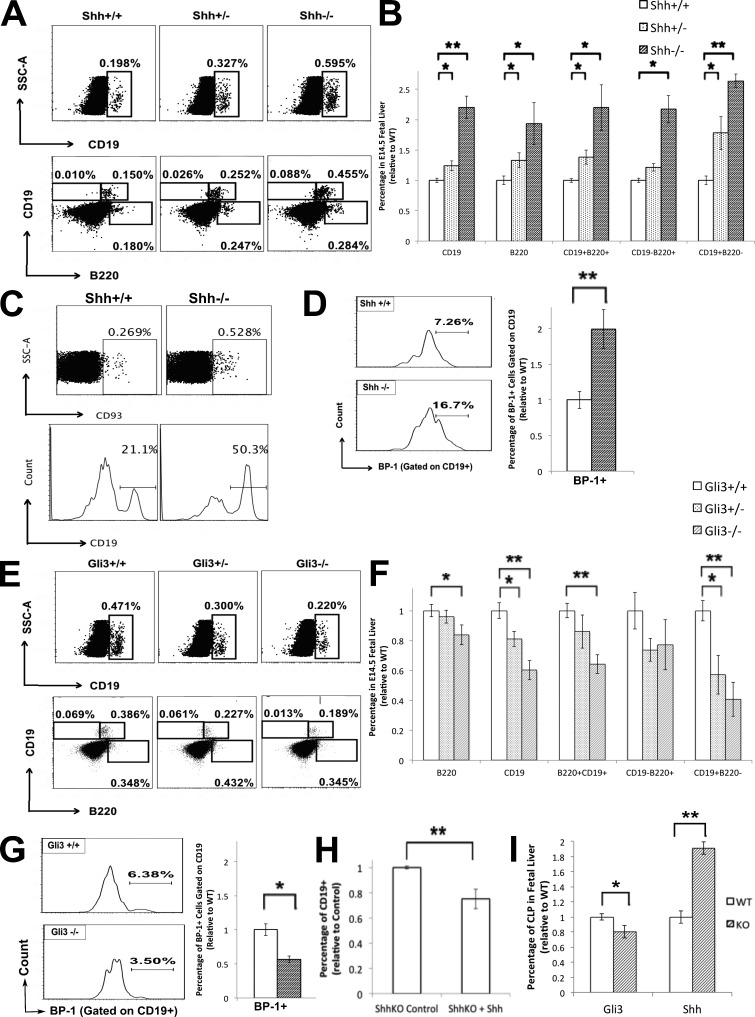
**B cell development in the E14.5 Shh-deficient and Gli3-deficient FL and Shh^−/−^ FLOC.** (A–D) Flow cytometry of E14.5 FLs from Shh^+/+^ (WT), Shh^+/−^, and Shh^−/−^ littermates. (A) Dot plots: SSC against CD19 staining (top) and CD19 staining against B220 staining (bottom). (B) Bar charts: relative mean percentage ± SEM, relative to the mean of WT littermates for Shh^−/−^ (*n* = 7), Shh^+/−^ (*n* = 10), and WT (*n* = 4) of populations stated, showing statistical significance compared with WT for CD19^+^ (Shh^+/−^, P = 0.03; Shh^−/−^, P = 0.002), B220^+^ (Shh^+/−^, P = 0.02; Shh^−/−^, P = 0.02), CD19^+^B220^+^ (Shh^+/−^, P = 0.03; Shh^−/−^, P = 0.02), CD19^−^B220^+^ (Shh^−/−^, P = 0.02), and CD19^+^B220^−^ (Shh^+/−^, P = 0.02; Shh^−/−^, P = 0.002) cells. (C, top) Dot plot: SSC against CD93 staining. (Bottom) histogram: CD19 staining gated on CD93. Both populations are statistically significant, relative to WT (P = 0.05 and P = 0.03, respectively). (D) Histogram: BP-1 staining gated on CD19. Bar chart: mean percentage ± SEM of this population, showing statistical significance relative to the mean of WT littermate for Shh^−/−^ (P = 0.003). (E and F) Flow cytometry of E14.5 FL from Gli3^+/+^ (WT), Gli3^+/−^, and Gli3^−/−^ littermates. For Shh^−/−^, *n* = 7; WT, *n* = 4. (E) Dot plots show SSC against CD19 staining (top) and CD19 staining against B220 staining (bottom). (F) Bar charts show the relative mean percentage ± SEM, relative to the mean of WT littermates of the populations stated, showing statistical significance relative to WT for B220^+^ (Gli3^−/−^, P = 0.04), CD19^+^ (Gli3^+/−^, P = 0.04; Gli3^−/−^, P = 0.003), CD19^+^B220^+^ (Gli3^−/−^, P = 0.003), CD19^−^B220^+^ (not significant), and CD19^+^B220^−^ (Gli3^+/−^, P = 0.03; Gli3^−/−^, P = 0.003). For Gli3^+/−^, *n* = 10; Gli3^−/−^, *n* = 9; Gli3^+/+^, *n* = 7. (G) Histogram: BP-1 staining gated on CD19. Bar chart: mean percentage ± SEM of this population, showing statistical significance relative to the mean of WT littermates for Gli3^−/−^ (P = 0.03). For Gli3^−/−^, *n* = 9; Gli3^+/+^, *n* = 7. (H) Shh^−/−^ FLOCs were treated with rShh for 4 d. Bar chart: mean (relative to mean of control cultures) percentage ± SEM of CD19^+^ cells. The difference was statistically significant by Student’s *t* test (P = 0.008; *n* = 4). (I) Bar charts: mean percentage ± SEM of CD117^+^CD127^+^ CLP cells in E17.5 Gli3^−/−^ (*n* = 9; P = 0.02) and E14.5 Shh^−/−^ (*n* = 4; P = 0.002) FLs, relative to their respective WT littermates. Shaded bars are knockout, and unshaded bars are WT. *, P ≤ 0.05; **, P ≤ 0.01.

To test whether Shh negatively regulates B lineage development in vivo, we assessed B cell development in Shh^−/−^ FLs. Most Shh^−/−^ embryos die before E16, so we analyzed the E14.5 FL. Both Shh^−/−^ and Shh^+/−^ had significantly increased percentages of CD19^+^ cells, B220^+^ cells, and of the CD19^+^B220^+^, CD19^−^B220^+^, and CD19^+^B220^−^ populations compared with WT, with the heterozygote showing intermediate proportions (Fig. 4, A and B). The proportion of B lineage–committed CD93^+^ cells and the proportion of CD93^+^ cells that were CD19^+^ were significantly increased in the Shh^−/−^ FL compared with WT (Fig. 4 C). The proportion of CD19^+^BP1^+^ (Pre-B) cells was also significantly increased in the Shh^−/−^ E14.5 FL compared with WT (Fig. 4 D).

As expected, the E14.5 *Gli3*-mutant FL showed the opposite phenotype, with significantly decreased CD19^+^, B220^+^, CD19^+^B220^+^, CD19^+^B220^−^, and CD19^+^BP1^+^ populations compared with WT littermates (Fig. 4, E–G). Treatment of E14.5 Shh^−/−^ FLOCs with rShh for 4 d reduced the proportion of CD19^+^ cells compared with the untreated control FLOCs (Fig. 4 H), confirming that Shh negatively regulates B cell development.

As Shh inhibited B cell development in B-committed populations, we investigated the impact of Shh deletion and Gli3 deletion on the development of CLPs. We analyzed the proportion of CLPs, defined by the surface markers Lin^−^CD117^+^CD127^+^, and found a significant increase in the CLP population in the Shh-deficient FL and decrease in the Gli3-deficient FL, relative to their WT littermates (Fig. 4 I). Collectively, our experiments indicate that Gli3 and Shh influence B cell development from the CLP populations through to the CD19^+^B220^+^pre-BCR^+^ stage of development.

### Conditional deletion of *Shh* from the hematopoietic compartment of the FL does not increase B cell development

**Figure 5.**
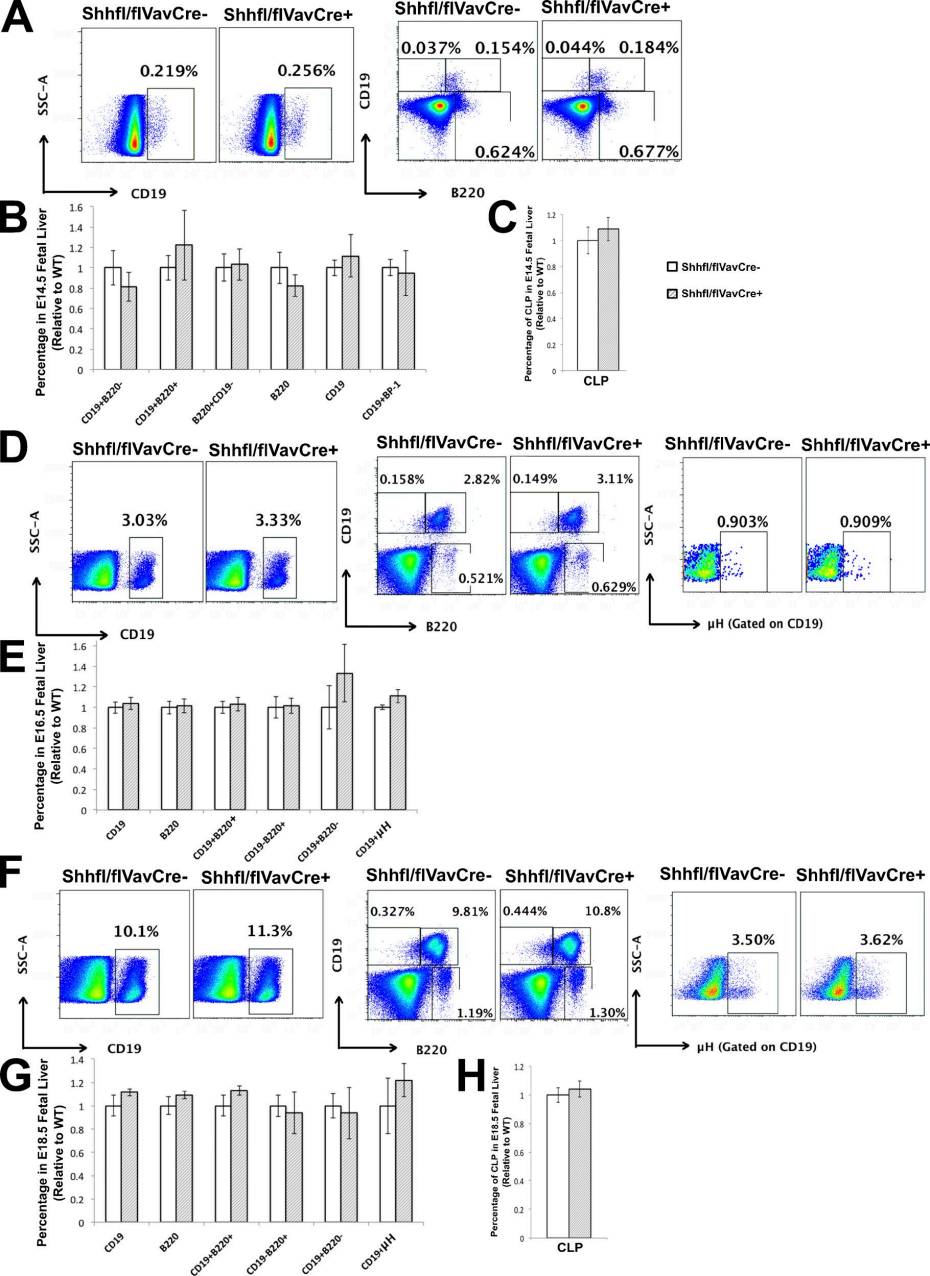
**B cell development in the *Shh^fl/fl^*VavCre FL.** (A–H) Flow cytometry of the E14.5 FL (A–C), E16.5 FL (D and E), and E18.5 FL (F–H) from *Shh^fl/fl^*VavCre^−^ (E14.5, *n* = 5; E16.5, *n* = 3; E18.5, *n* = 5) and *Shh^fl/fl^*VavCre^+^ (E14.5, *n* = 3; E16.5, *n* = 5; and E18.5, *n* = 3) littermates. (A, D, and F) Dot plots: SSC against CD19 staining (left), CD19 staining against B220 staining (middle), and µH staining for D and F (right). (B, E, and G) Bar charts: mean percentage, relative to mean of WT littermates ± SEM of the B220^+^, CD19^+^, CD19^+^B220^+^, CD19^−^B220^+^, CD19^+^B220^−^, CD19^+^BP-1^+^, and CD19^+^µH^+^ populations. (C and H) Bar charts: mean percentage ± SEM of Lin^−^CD117^+^CD127^+^ CLPs. There were no significant differences by Student’s *t* test. Shaded bars are Cre^+^, and unshaded bars are Cre^−^.

To investigate whether the increase in B cell development in the E14.5 Shh^−/−^ FL is caused by cell-intrinsic loss of Shh expression in the hematopoietic compartment or caused by loss of Shh secretion by the nonhematopoietic compartment (stroma), we used the Cre-loxP system to conditionally delete *Shh* from the hematopoietic lineage in *Shh^fl/fl^*VavCre^+^ (ShhcoKO) embryos. We found no significant differences in B cell differentiation between the control (Cre negative) and ShhcoKO E14.5 FL and no significant differences in the proportions of cells that expressed CD19, B220, and BP-1 and in the proportion of CLPs between control and ShhcoKO (Fig. 5, A–C). We likewise found no significant differences in B cell populations defined by cell surface expression of CD19, B220, and µH on E16.5 and E18.5 and no significant difference in the proportion of CLPs on E18.5 between ShhcoKO and WT (Fig. 5, D–H). Therefore, it is *Shh* expression by the FL stroma, rather than hematopoietic cell–intrinsic Shh expression, that regulates B cell differentiation. This is consistent with the increase in Shh expression observed in the Gli3^−/−^ FL stroma compared with WT (Fig. 2 H) and with the fact that conditional deletion of Gli3 from the hematopoietic compartment has no influence on B cell differentiation (Fig. 1, J–L). Collectively, these experiments indicate that Gli3 activity in the FL stroma promotes B cell development by repression of Shh expression in the stroma.

### *Gli3* mutation inhibits transcription of B cell–lineage commitment, signaling, and maturation genes

**Figure 6.**
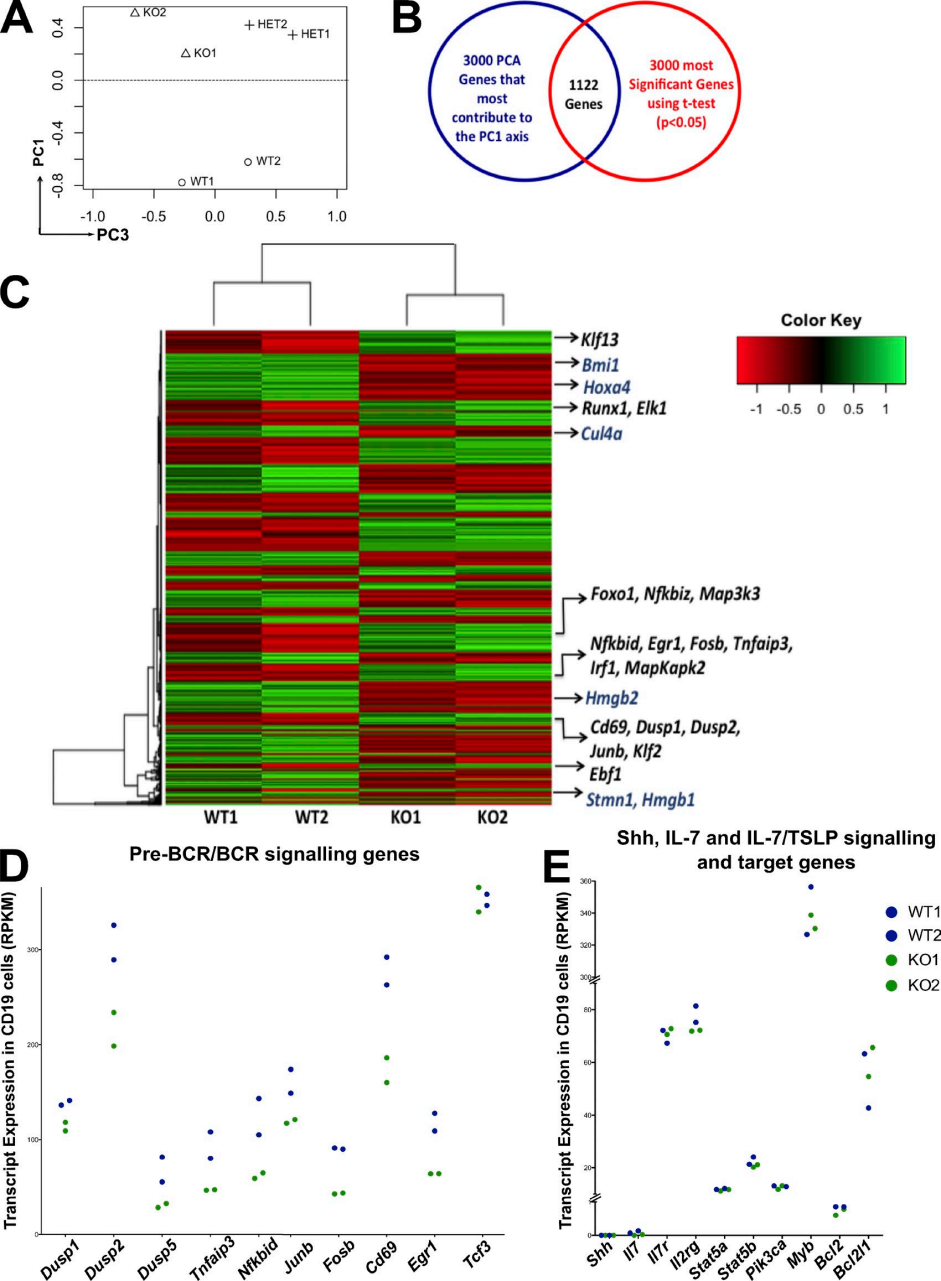
**RNA sequencing detects transcriptional differences in Hh signaling genes and B cell differentiation and signaling genes between the WT and *Gli3*-mutant CD19^+^B220^+^ cells.** (A) PCA showing sample relationships in PC1 and PC3 for WT (*n* = 2), Gli3^+/−^ (*n* = 2), and Gli3^−/−^ (*n* = 2) CD19^+^B220^+^ populations from E17.5 FLs. (B) Venn diagram: 1,122 genes intersect out of the 3,000 genes that contributed most to PC1 (highest and lowest scoring genes) and the 3,000 most significant differentitally expressed genes by Ebayes statistics. (C) Gene expression heat map showing Hh signaling genes in blue and B cell differentiation and signaling genes in black. Normalized expression signals are represented as a z score where green is lower expression and red is higher expression levels. (D and E) Transcript expression (reads per million kilobases) of pre-BCR/BCR signaling and target genes (D) and Shh, IL-7, and IL-7/thymic stromal lymphopoietin (TSLP) signaling and target genes (E) in the Gli3^−/−^ (green; *n* = 2) and WT (blue; *n* = 2) RNA sequencing datasets.

To investigate the mechanisms of action of Gli3 on B cell development, we measured transcription in developing B cells in the *Gli3*-mutant FL. We used RNA sequencing to analyze whole-genome expression in FACS-sorted CD19^+^B220^+^ FL B cells in the WT and *Gli3* mutants. First, we analyzed the dataset in an unbiased manner using principal component analysis (PCA). The dataset segregated by genotype on both principal component axis 1 (PC1) and PC3 (Fig. 6 A). PC1, the axis attributing to the largest differences in the dataset (60% variability), separated the WT from the *Gli3* mutants (Gli3^+/−^ and Gli3^−/−^), whereas PC3 showed differences between Gli3^+/−^ and Gli3^−/−^ (Fig. 6 A).

Further analysis of the genes with high positive and negative scores on PC1 showed that PC1 reflected differences in genes associated with Hh signaling and genes associated with B cell signaling and differentiation. Key Hh signaling and target genes, including *Gli1*, *Hdac3*, and *Smo*, had positive PC1 scores, indicating that the expression of these is higher in the *Gli3* mutant. In contrast, genes that were lower in the *Gli3* mutants had high negative PC1 scores and were mainly B cell–signaling and –lineage commitment genes. Thus, not only did *Gli3* mutation reduce the proportion of CD19^+^B220^+^ B-committed cells in the FL, but also, within that sorted population, expression of genes required for B cell differentiation was reduced.

To understand better the genome-wide differences in the dataset, we intersected the differentially expressed genes identified by Ebayes statistics with the genes identified by PCA. We selected the 3,000 genes (highest and lowest scorers) that contributed most to the PC1 axis and intersected these with the 3,000 most significantly differentially expressed genes by Ebayes statistics (Table S1). The resulting 1,122 genes were clustered, and a heat map showing their gene expression was drawn (Fig. 6, B and C). This intersection highlighted genes that have been previously shown to be *Shh*-target genes in other tissues, such as *Stmn1*, *Hmgb1*, *Hmgb2*, *Hoxa4*, *Cul4a*, and *Bmi1*, which were all up-regulated in the *Gli3* mutant (Fig. 6 C; Itou et al., 2011; Wang et al., 2012; Lu et al., 2015; Yang et al., 2015). In contrast, master regulators of early B cell development *Ebf1* and *Foxo1* were down-regulated in the *Gli3* mutants compared with WT, and several other B lineage differentiation and maturation genes, including *Klf13*, *Egr1*, *Irf1*, *Irf4*, and *Cd69*, were also lower in the *Gli3* mutant (Gururajan et al., 2008; Lu, 2008; Outram et al., 2008). B cell activation and signaling genes including genes involved in modulating MAPK signaling such as *Dusp1*, *Dusp2*, *Map3k3*, *MapKapk2* (Lang et al., 2006), and canonical NF-κB signaling genes (*Nfkbid*, *Nfkbiz*, and *Tnfaip3*) were all down-regulated in the *Gli3* mutant. In addition, the AP1 components *Fosb*, *Jun*, and *Junb*, required for pre-BCR signal transduction (de Gorter et al., 2007), and *Klf2*, an essential late target gene of the pre-BCR, were also lower in the *Gli3* mutant (Winkelmann et al., 2014).

As both pre-BCR/BCR signaling and IL-7 signaling are important regulators of B cell development, we compared expression levels of genes that encode downstream components of these signaling pathways or are their immediate transcriptional targets (Fig. 6 D). Expression levels of many genes involved in pre-BCR/BCR signal transduction (*Dusp1-3*, *Tnfaip3*, *Nfkbid*, and the AP-1 components *Junb* and *Fosb*) were lower in the Gli3^−/−^ datasets than WT, as were its immediate transcriptional targets *CD69* and *Egr1* (Fig. 6 D). We observed no difference in expression in *Tcf3* (E2A) between genotypes (Fig. 6 D) or in *IL7r*, *Il2rg*, *Stat5a*, *Stat5b*, and *Pik3ca* (components of the IL-7/thymic stromal lymphopoietin signaling pathway) or in *Myb*, *Bcl2*, or *Bcl2l1* (transcriptional targets of IL-7 signaling in B-lineage cells; Fig. 6 E).

We did not detect *Shh* expression in any dataset (Fig. 6 E), consistent with the Q-RT-PCR showing *Shh* up-regulation in Gli3^−/−^ FLs being restricted to stromal cells and not B-lineage cells (Fig. 2 H) and with the fact that conditional deletion of *Shh* from B-lineage cells had no impact on their development (Fig. 5).

Together these analyses indicate that the *Gli3*-mutant B220^+^CD19^+^ population has increased Hh-dependent transcription, consistent with our previous results showing increased *Shh* transcription in the *Gli3*-mutant FL stroma (Fig. 2 H) and increased Hh signaling in Gli3^−/−^ B-lineage cells (Fig. 2, G, I–K, and M). Gli3 deficiency also decreased transcription of genes required for B cell differentiation, maturation, and signaling within the sorted B220^+^CD19^+^ population. Thus, increased Shh signaling reduced transcription of regulators of B lineage commitment and differentiation. Therefore, we investigated whether Shh treatment can directly down-regulate transcription of the key B-lineage transcription factors *Ebf1* and *Pax5* in vitro.

### Shh signaling leads to reduced *Ebf1* and Pax5 expression during B lineage development

Ebf1 and Pax5 are master regulators of B-lineage commitment and B cell development. B cell–lineage commitment from the CLP stage is regulated by Ebf1 (Zhang et al., 2003), which promotes its own transcription as well as *Pax5* transcription. Pax5 further increases *Ebf1* transcription (Roessler et al., 2007) creating a positive feedback loop between itself and Ebf1. This mechanism allows Ebf1 and Pax5 to regulate B cell development and maturation. Therefore, we tested whether the increase in *Shh* in the *Gli3* mutant influences *Ebf1* and *Pax5* expression during B cell development.

**Figure 7.**
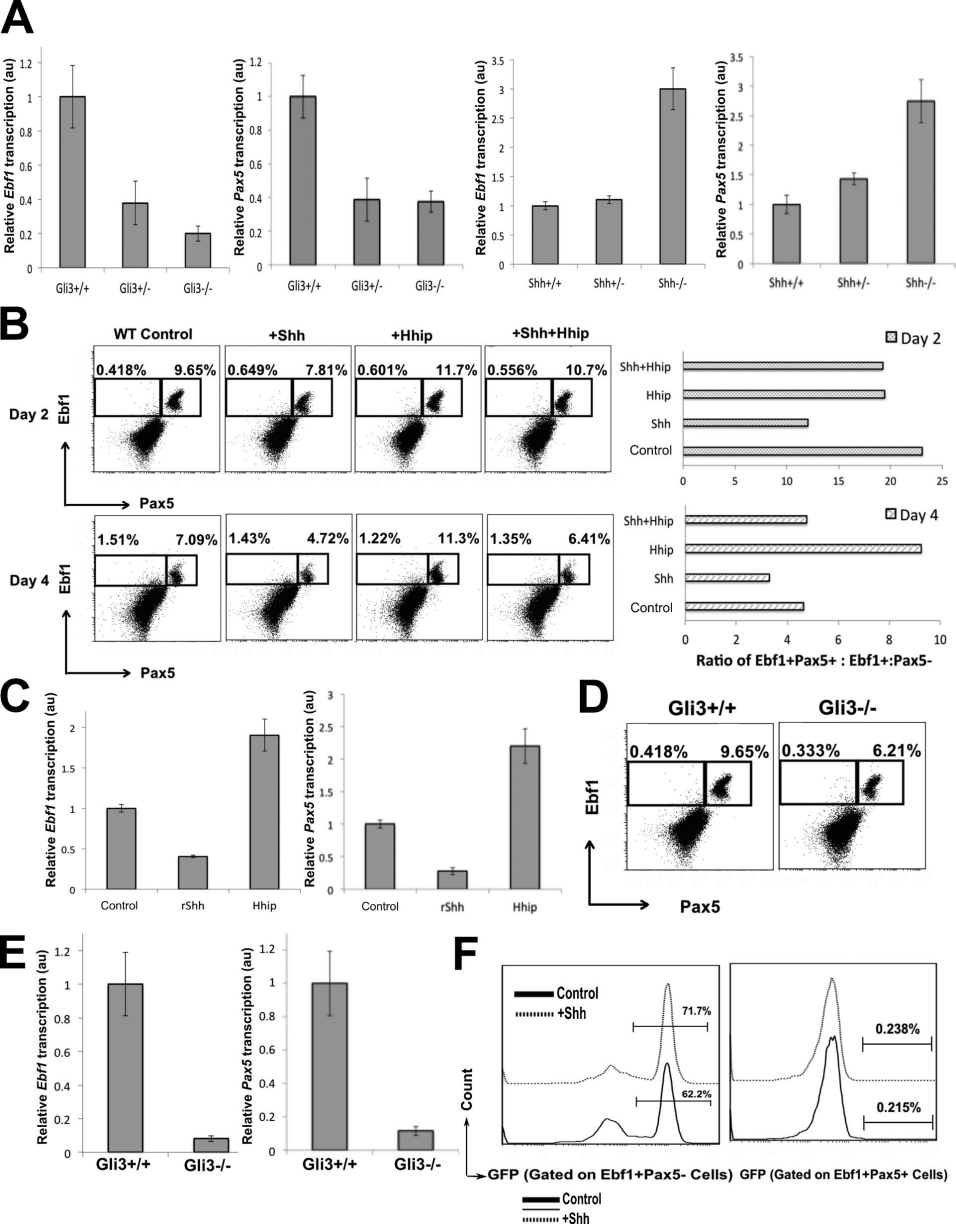
**Expression of Pax5 and Ebf1 in the FL.** (A) Representative experiment showing mean ± SEM (*n* = 3) of Q-RT-PCR for *Pax5* and *Ebf1* in FLs from *Gli3* and *Shh* mutants compared with WT littermates. au, arbitrary units. (B and C) WT FLOCs (*n* = 3) were treated with rShh, rHhip, and rShh + rHhip for 2 and 4 d, compared with untreated control cultures, and analyzed by flow cytometry and Q-RT-PCR. (B) Dot plots: anti-Ebf1 and anti-Pax5 staining on day 2 (top) and day 4 (bottom). Charts: ratio of Ebf1^+^Pax5^+^ to Ebf1^+^Pax5^−^ cells in the different culture conditions on day 2 (top) and day 4 (bottom). (C) Bar charts: representative experiment showing mean ± SEM (*n* = 3) of Q-RT-PCR for *Pax5* and *Ebf1* on day 4 of WT FLOCs treated with rShh or rHhip and control untreated. (D) Dot plots: anti-Pax5 and anti-Ebf1 staining in Gli3^+/+^ and Gli3^−/−^ littermate FLs. (E) Representative experiment showing mean ± SEM (*n* = 3) of Q-RT-PCR for *Pax5* and *Ebf1* in purified CD19^+^ cells from Gli3^+/+^ and Gli3^−/−^ E17.5 FLs. All transcript expression levels were normalized relative to *HPRT*. (F) Histograms: GFP expression in Ebf1^+^Pax5^−^ and Ebf1^+^Pax5^+^ cells in the E17.5 GBS-GFP–transgenic FLOCs, control (continuous lines), and rShh treated (dotted lines) FLs, cultured for 2 d.

First, we measured overall *Ebf1* and *Pax5* transcription in the *Gli3*-mutant E17.5 FL and found a reduction in both Gli3^+/−^ and Gli3^−/−^ relative to WT (Fig. 7 A), consistent with the RNA sequencing data and the reduction in B cell development. In contrast, we found an increase in the expression of both transcription factors in the Shh^+/−^ and Shh^−/−^ E14.5 FL, consistent with increased commitment to the B lineage in the absence of Shh (Fig. 7 A).

Because the *Gli3* mutant has increased Shh signaling and rShh treatment decreased the CD19^+^ population in FLOCs, we tested whether we could influence both *Pax5* and *Ebf1* transcription and protein expression in vitro by modulating Hh signaling in FLOCs by treatment with rShh or rHhip over a 4-d culture period. We measured the expression of intracellular Pax5 and Ebf1 by FACS analysis to investigate protein expression in single cells. On day 1 after treatment, there were no significant differences in the proportions of Ebf1^+^Pax5^−^ and Ebf1^+^Pax5^+^ cells (not depicted). However, a significant reduction in the proportion of Ebf1^+^Pax5^+^ cells was seen on days 2 and 4 of rShh treatment (Fig. 7 B). This Ebf1^+^Pax5^+^ population significantly increased and was sustained in the rHhip-treated cultures, whereas the cultures in which both rShh and rHhip were added together were not different from the control cultures (Fig. 7 B). Comparison of the ratio of Ebf1^+^Pax5^+^ cells to Ebf1^+^Pax5^−^ cells showed that rHhip treatment increased the ratio by twofold by day 4 in culture, indicating that inhibition of Hh signaling accelerated the transition from Ebf1^+^Pax5^−^ cell to Ebf1^+^ Pax5^+^ cell. In contrast, rShh treatment reduced this ratio on both days 2 and 4 of culture, suggesting that Shh signaling repressed the induction of Pax5 and the transition to Ebf1^+^Pax5^+^ cell. The changes in the Ebf1 and Pax5 proteins were consistent with the changes in transcription of *Ebf1* and *Pax5* on day 4 (Fig. 7 C). In the Gli3^−/−^ FL, the proportion of Ebf1^+^Pax5^+^ cells was reduced compared with WT, consistent with the Q-RT-PCR data (Fig. 7 D).

Then, we FACS sorted CD19^+^ cells from Gli3^−/−^ and WT littermates and analyzed the expression of *Ebf1* and *Pax5*. We observed a decrease in both *Ebf1* and *Pax5* transcription in purified CD19^+^ cells from the *Gli3*-mutant FL compared with WT (Fig. 7 E). Interestingly, the decrease in expression of both *Ebf1* and *Pax5* in the sorted Gli3^−/−^ CD19^+^ cells was more pronounced than the reduction in transcript expression in the unsorted FL and was also greater than the proportional change in the CD19^+^ population in the Gli3^−/−^ FL compared with WT. This was consistent with the action of Shh to reduce B cell development in FLOCs, in which the magnitude of the reduction in *Pax5* and *Ebf1* transcription was greater than the change in the Ebf1^+^Pax5^+^ population (Fig. 7, B and C) and also greater than the magnitude of the change in the overall proportion of B-lineage cells caused by Shh treatment (Fig. 3 A). Thus, Shh treatment in vitro or Gli3 deficiency in vivo not only reduced the proportion of CD19 cells that were present, but also led to a reduction in transcription of the key B-lineage master regulators *Ebf1* and *Pax5* within the B lineage–committed population, most likely by signaling to up-regulate the transcription of an intermediate transcriptional repressor of *Ebf1* and/or *Pax5*.

To investigate the relationship between Hh-mediated transcription and the reduction in transcription of *Ebf1* and *Pax5*, we measured intracellular Ebf1 and Pax5 expression in rShh-treated and control untreated GBS-GFP–transgenic FLOCs. We found that >62% of Ebf1^+^Pax5^−^ cells expressed high levels of GFP, indicating that this population actively mediates Hh-dependent transcription, which is therefore compatible with Ebf1 protein expression (Fig. 7 F). These cells are the earliest B cell population that differentiates from the CLP stage (Egawa et al., 2001; Mebius et al., 2001). Interestingly, GFP expression was reduced to <0.3% in the later Ebf1^+^Pax5^+^ population (Fig. 7 F), indicating that Hh signaling decreases as cells become more mature and that, once Pax5 is expressed, very few cells are undergoing Hh-mediated transcription. This is consistent with Shh signaling acting directly or indirectly to inhibit Pax5 transcription. The Ebf1^+^Pax5^−^ population was Hh responsive, as expression of GFP in the Ebf1^+^Pax5^−^ population was increased on rShh treatment, with the proportion of GFP-negative cells decreasing from ∼38% to ∼28%. In contrast, GFP was not induced by rShh treatment in the Pax5^+^Ebf1^+^ population during the 2-d culture, and the proportion of GFP-negative cells remained >99.7% under both conditions.

Collectively, our experiments indicate that the *Gli3*-mutant FL had increased Hh signaling resulting in decreased B cell development. Furthermore, loss of Shh in vitro and in vivo led to increased B-lineage commitment and development. We propose that Shh signaling either directly or indirectly represses Pax5 expression (most likely by transcriptional activation of a transcriptional repressor of *Pax5*) and that this leads to loss of Pax5’s induction of Ebf1, reduction in both Pax5 and Ebf1 expression, and therefore to the negative regulation of the B lineage development observed.

## Discussion

Here, we showed that Gli3, expressed by the stromal compartment, is required for B cell development in the FL. Mutation of *Gli3* led to an overall reduction in B lineage–committed cells, reduction in the proportion of pre–B cells, and reductions in CLPs and both the CD19^+^B220^−^ B1 progenitor population on E14.5 and the CD19^−^B220^+^ B2 progenitor population on E17.5. *Gli3* mutation additionally reduced expression of B lineage–specifying and –signaling genes within the B220^+^CD19^+^ population, indicating that the effect of *Gli3* mutation is not entirely caused by its influence on CLPs and the earliest B cell progenitors but that it continues to influence the more mature B lineage–committed population. The changes in B cell differentiation in the *Gli3*-mutant FL could be caused by Hh-dependent or Hh-independent effects. We found that loss of Gli3 led to increased *Shh* expression and overall increased Hh signaling in the mouse FL. Thus, Gli3 was acting as a repressor of the Hh pathway in the FL, as observed in the development of other cells and tissues, such as the neural stem cells, vertebrate limb bud, and thymus (te Welscher et al., 2002; Hager-Theodorides et al., 2009; Petrova et al., 2013; Saldaña et al., 2016). Treatment of Gli3-deficient FLOCs with rHhip (to neutralize endogenous Hh proteins) increased B cell development, and therefore, the reduction in B cell development in the Gli3 mutants was Hh dependent and caused by increased Shh expression in the Gli3^−/−^ FL.

In contrast, the Shh-deficient FL had increased B lineage commitment and B cell differentiation, demonstrating that Shh negatively regulates B cell development in vivo. We showed that both *Shh* transcription in FL stroma and Hh signaling to B-lineage cells are increased in the Gli3-deficient FL and that Shh negatively regulates B cell development by signaling directly to developing hematopoietic cells, as Hh-target genes were up-regulated in the Gli3^−/−^ CD19^+^B220^+^ population, and the Hh-reporter transgenic FL showed increased GFP expression in the Gli3^−/−^ CD19^+^ population.

Expression of the master regulators of B cell development, Ebf1 and Pax5, was reduced in the Gli3-deficient FL but increased in the Shh-deficient FL, and treatment of WT FLOCs with Hhip to neutralize endogenous Hh molecules in the cultures increased the proportion of Ebf1^+^Pax5^+^ cells and increased transcription of both *Ebf1* and *Pax5* within the CD19^+^ population, whereas rShh treatment had the opposite effect. The Ebf1^+^Pax5^−^ population showed high Hh-mediated transcription in the Hh-reporter transgenic FL, and therefore, we proposed that Shh signaling within the Ebf1^+^Pax5^−^ cells reduced expression of Pax5, thereby also reducing the Pax5-dependent induction of Ebf1 expression, leading to an overall reduction in B cell development.

Shh signaling from follicular dendritic cells to B cells in the adult spleen has been shown to promote B cell survival and function (Sacedón et al., 2005), and in the adult BM, components of the Hh signaling pathway are expressed in developing B cells (Heng et al., 2008; Cooper et al., 2012). However, conditional deletion of *Smo* from the B cell lineage did not influence B cell development in the adult BM (Cooper et al., 2012). Our study demonstrates that the Hh signaling pathway is active in developing fetal B cells and regulates B cell development in the FL. Therefore, there may be tissue- or life stage–specific differences in the function of Hh signaling between FL and adult BM. Alternatively, it is possible that Hh pathway activation is noncanonical (Smo independent) in B-lineage cells, which do not have primary cilia, or that a balance between canonical and noncanonical signaling may exist, as described in osteoblast differentiation (Yuan et al., 2016).

Our genome-wide RNA sequencing data from the sorted CD19^+^B220^+^ population in the *Gli3* mutants revealed many differentially expressed genes between Gli3^−/−^ and WT. Many Hh-target genes (e.g., *Stmn1*, *Hdac3*, *Hoxa4*, *Hmgb2*, *Bmi1*, and *Cul4a*) were up-regulated in the *Gli3* mutants, consistent with the increased Hh-mediated transcription measured using the GBS-GFP reporter and confirming that Shh signals directly to developing B cells.

In contrast, many B cell signaling pathway genes involved in NF-κB activity (*Nfkbid*, *Nfkbiz*, and *Tnfaip3*), MAPK signaling (*MapKapk2* and *Map3k3*), and components of AP-1 (*Junb* and *Fosb*) were decreased in the *Gli3* mutant. These pathways are required for pre-BCR and BCR signaling (Feng et al., 2004; de Gorter et al., 2007). In addition, *Tnfaip3* regulates marginal zone and B1 cell development in the adult (Chu et al., 2011), and we observed a reduction in B1 progenitor cells in the E14.5 Gli3^−/−^ FL. Interestingly, Hh-mediated transcription in developing and mature T cells also represses expression of genes that regulate activity of NF-κB, MAPK, and AP1, leading to reduced pre-TCR and TCR signaling (Rowbotham et al., 2007, 2009; Furmanski et al., 2012, 2015; Barbarulo et al., 2016).

In addition to the decreased transcription of genes associated with pre-BCR and BCR signaling, the RNA sequencing revealed a significant decrease in key transcriptional regulators of B cell differentiation including *Ebf1*, *Foxo1*, *Runx1*, and *Irf4* (Dengler et al., 2008; Niebuhr et al., 2013). *Ebf1* is required from the early CLP stage to the late mature stages of B cell development (Roessler et al., 2007; Nechanitzky et al., 2013) and, importantly, activates transcription of another key master regulator of B cell development, Pax5. Then, Pax5 promotes Ebf1 transcription, creating a positive feedback loop, which supports all stages of B cell development (Roessler et al., 2007).

We found high Hh pathway activity (measured by GFP expression in the Hh-reporter embryo) in Ebf1^+^Pax5^−^ cells, but GFP expression ceased in the next Ebf1^+^Pax5^+^ population. Both Ebf1 and Pax5 protein and gene expression were reduced by rShh treatment and increased by neutralization of Hh proteins by rHhip treatment in FLOCs, and manipulation of Hh signaling influenced the transition from Ebf1^+^Pax5^−^ to Ebf1^+^Pax5^+^ cell. Therefore, we propose that Shh signaling to developing B cells functions to reduce Pax5 expression, which then breaks the positive feedback loop, leading to reduction in Ebf1 expression. Interestingly, Shh signaling has been shown to interact with and regulate other Pax family members in the development of other tissues (Chi and Epstein, 2002; Blake and Ziman, 2014).

Dysregulated Hh pathway activation is involved in some B cell malignancies (Dierks et al., 2007; Lin et al., 2010), including B cell acute lymphoblastic leukemia (B-ALL; Ramirez et al., 2012; Qu et al., 2013), a common cancer of early childhood, and microarray expression profiles show that Hh pathway components are expressed in human FL and BM (Fig. S1 A; Su et al., 2004). Understanding the function of Hh signaling in normal fetal B cell development and its effect on Pax5 and Ebf1 expression will be important to our understanding of its role in B-ALL. In the future, it will be important to investigate how dysregulated Hh signaling influences Pax5 and Ebf1 activity in B-ALL.

In summary, we show that Gli3 activity in the FL stroma is required for normal B cell development. We showed that Shh signaling directly to B-lineage cells negatively regulates their development.

## Materials and methods

### Mice

C57BL/6 mice were purchased from Envigo. GBS-GFP–transgenic (GBS-GFP-tg) mice were provided by J. Briscoe (Crick Institute, London, England, UK; Balaskas et al., 2012), Vav-iCre-tg by D. Kioussis (National Institute for Medical Research, London, England, UK; de Boer et al., 2003), and Shh^+/−^ mice by P. Beachy (Stanford University School of Medicine, Palo Alto, California; Chiang et al., 1996) and were backcrossed for >12 generations on C57BL/6 mice. *Gli3^fl/fl^*, *Shh^fl/fl^*, and Gli3^+/−^ mice on C57BL/6 background were purchased from The Jackson Laboratory. Time mates were obtained by mating overnight, and the next day was counted as E0.5. Mice were bred and maintained at University College London under UK Home Office regulations, and experiments were approved by the University College London ethical approval committee.

### Flow cytometry, antibodies, and cell purification

FL cell suspensions were made by crushing each FL between two frosted slides. Where stated, red blood cells were lysed using 1× RBC lysis buffer (eBioscience) according to the manufacturer’s instructions. Cells were stained as described previously (Hager-Theodorides et al., 2005) using directly conjugated antibodies from BD, BioLegend, and eBioscience. Data were acquired on a C6 Accuri flow cytometer (BD) and analyzed using FlowJo software (Tree Star). Live cells were gated by forward scatter and side scatter (SSC) profiles. The data represent at least three experiments. In some experiments, CD19^+^ cells were purified using the EasySep biotin magnetic bead positive selection kit (STEMCELL Technologies) according to the manufacturer’s instructions, and RNA was extracted for Q-RT-PCR analysis.

### FLOCs

FLs were extracted from embryos at different stages of development. They were cut into ∼1-mm cubes and cultured on 0.8-µm filters (EMD Millipore) in 1 ml of AIM-V serum-free medium (Invitrogen) in 24-well plates for up to 4-d at 37°C and 5% CO_2_ before analysis. In some experiments, rHhip (Sigma-Aldrich) or rShh (R&D Systems) was added at 1 µg/ml. To allow comparison between litters for statistical analysis, relative numbers or percentages for each genotype or treatment were calculated by dividing by the mean of controls from the same litter (untreated control or WT littermates).

### RNA sequencing and data analysis

FL from WT, Gli3^+/−^, and Gli3^−/−^ embryos (*n* = 2) were dissected on E17.5 and crushed between two frosted slides. The cell suspension was stained using the antibodies CD19-APC and B220-PeCy7, and the double-positive B220^+^CD19^+^ population was FACS sorted. RNA from this population was extracted using an Arcturus PicoPure RNA Isolation kit (Applied Biosystems), and quantity and quality were determined by a Bioanalyzer 2100 (Agilent Technologies).

RNA was sequenced by University College London Genomics on a NextSeq 500 system (Illumina). The sequenced data are publically available in the GEO database under accession no. GSE81467. The RNA sequencing dataset was processed and standardized using the Bioconductor package DESeq2. The Bioconductor package DESeq2 was used to generate normalized estimates of transcript abundance, expressed as RPKM (reads per kilobase of transcript per million mapped reads). Differentially expressed genes were determined using the moderated Ebayes t statistic P < 0.05 from the limma package in Bioconductor. PCA was performed using the CRAN package ade4.

### Q-RT-PCR

RNA extraction and cDNA synthesis were performed as described previously (Sahni et al., 2015). We used the QuantiTect primers for *Gli1*, *Gli2*, *Shh*, *Hhip*, *Gli3*, *Smo*, *Ptch1*, *Ebf1*, and *Pax5* from QIAGEN. The cDNA samples were prepared using the iQ SYBR Green Supermix (Bio-Rad Laboratories) according to the manufacturer’s instructions and run on a iCycler system (Bio-Rad Laboratories). Gene transcript levels were normalized relative to *HPRT*.

For quantification of VH to JH rearrangements, we prepared RNA from FACS-sorted CD19^+^ cells from Gli3^−/−^ and WT E17.5 FL and followed the protocol described by Braikia et al. (2014), using the primers combinations: VH7183-Fw and JH1R; VHJ558-Fw and JH1R; and HS5-Fw1 and HS5-R1 for normalization.

### PCR analysis for genotyping

DNA for PCR analysis was extracted from tissues by digesting in lysis buffer containing 50 mM KCl, 1.5 mM MgCL_2_, 10 mM Tris HCL, pH 8.5, 0.01% gelatin, 0.45% Nonidet P-40, 0.45% Tween 20, and 0.5 µg/ml proteinase K (Sigma-Aldrich) in water. Approximately 1 µg DNA was used as a template in each PCR reaction, using primers for *Shh^−/−^*, *Shh^+/−^*, and *Shh^+/+^* as described by Shah et al. (2004). Gli3^+/+^, Gli3^+/−^, and Gli3^−/−^ were distinguished phenotypically (Johnson, 1967), and genotype was confirmed by PCR as previously described (Hager-Theodorides et al., 2005). *Gli3^fl/fl^*, *Shh^fl/fl^*, and Vav-iCre-tg mice were genotyped as previously described (Saldaña et al., 2016).

### Statistical analysis

Statistical analysis was performed using unpaired two-tailed Student’s *t* tests, and probabilities were considered significant if P ≤ 0.05 (*), P ≤ 0.01 (**), or P ≤ 0.001 (***).

### Online supplemental material

Fig. S1 represents the transcript expression of Hh pathway members (*GLI1*, *GLI2*, *GLI3*, *PTCH1*, *SMO*, and *SHH*) in the human BM and FL and shows transcript expression of Hh molecules (*Shh*, *Ihh*, and *Dhh*) and pathway components *Gli1*, *Gli2*, *Gli3*, *Ptch1*, and *Smo* from the ImmGen database in mouse FL. Table S1 is available as an Excel file and contains a list of 3,000 differentially expressed genes, significant by Ebayes statistics, 1,500 genes with high positive PC1 scores, and 1,500 genes with high negative PC1 scores.

## Supplementary Material

Supplemental Materials (PDF)

Table S1 (Excel file)
