# Supplementary material for: The transcription factor Gli3 promotes B cell development in fetal liver through repression of Shh
Source: J Exp Med. 2017 Jul 3;214(7):2041–58. doi: 10.1084/jem.20160852 (PMC5502423; doi:10.1084/jem.20160852)
Supplement: Supplemental Materials (PDF) [file JEM_20160852_sm.pdf]

The Journal of Experimental Medicine

SUPPLEMENTAL MATERIAL

Solanki et al., https://doi.org/10.1084/jem.20160852

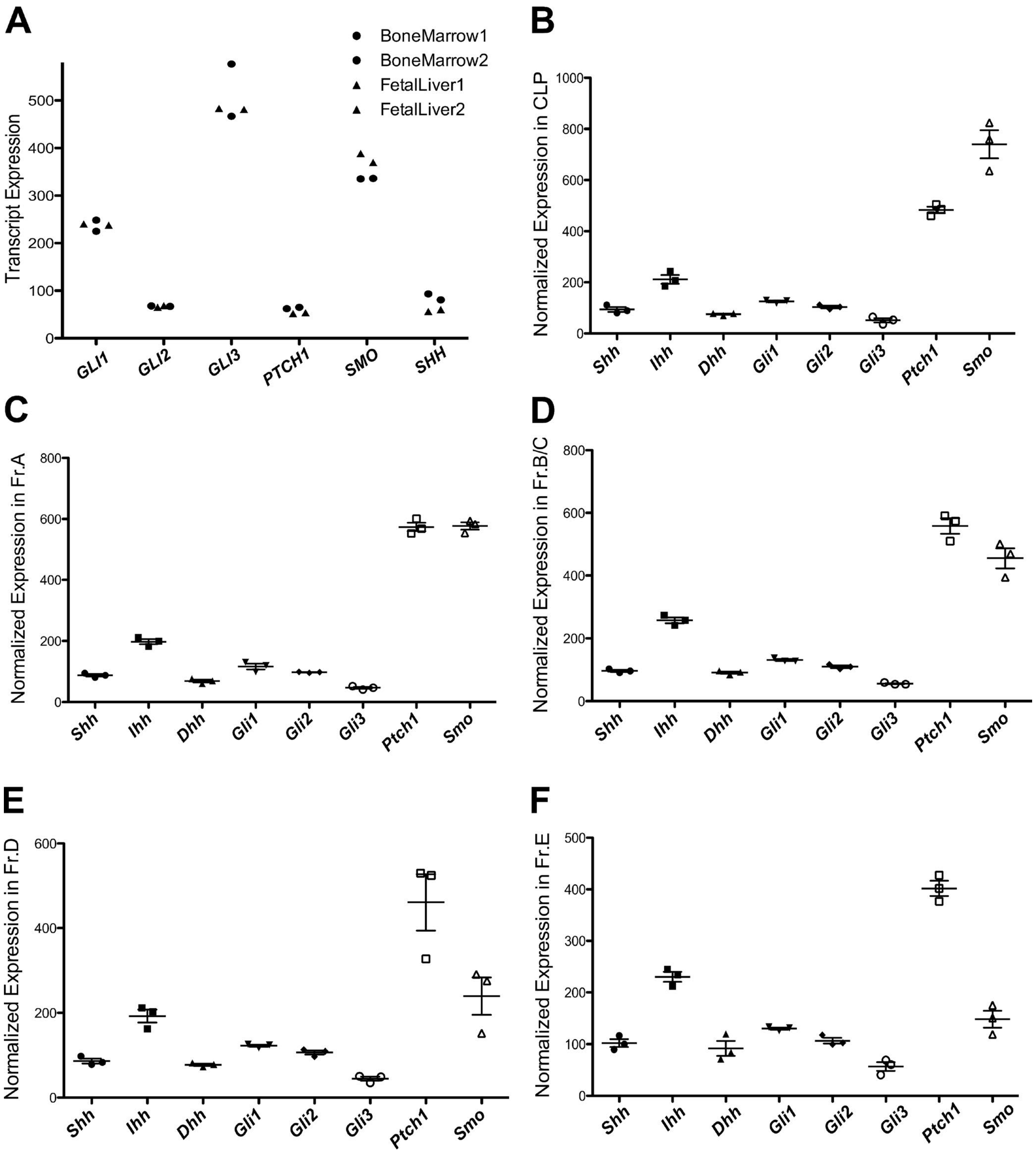

Figure S1. **Expression of Hh ligands and Hh signaling pathway components in human FL and BM and mouse B cell FL populations.** (A) Transcript expression of human Hh pathway members (*GLI1*, *GLI2*, *GLI3*, *PTCH1*, *SMO*, and *SHH*) in the human BM (circles) and FL (triangles) for duplicate biologically independent microarrays from the publically available GEO database (accession no. GSE1133). (B–F) Transcript expression of (*Shh*, *Ihh*, *Dhh*, and *Gli1*, *Gli2*, *Gli3*, *Ptch1*, and *Smo*) in CLP ($AA4^+Kit^+IL\text{-}7ra^+B220^-$; B), fraction A (Fr.A; $AA4^+Kit^+IL\text{-}7ra^+B220^+$; C), fraction B/C ($AA4^+IgM^-CD19^+CD43^+HSA^+$; D), fraction D ($AA4^+IgM^-CD19^+CD43^-HSA^+$; E), and fraction E ($AA4^+IgM^+CD19^+HSA^+$; F). Triplicates are from microarrays from independent biological replicates from the Immgen database (GEO database accession no. GSE15907). Line shows mean normalized expression ± SEM for each gene.

Table S1 is available as an Excel file and contains a list of 3,000 differentially expressed genes, significant by Ebayes statistics, 1,500 genes with high positive PC1 scores, and 1,500 genes with high negative PC1 scores.
